# Posterior malleolus fracture: a mid-term follow-up

**DOI:** 10.1186/s13018-022-03488-5

**Published:** 2023-01-04

**Authors:** Yuan Quan, Hao Lu, Peng Qi, Songyao Tian, Jiantao Liu, Chunlong Zhang, Boyu Zhang, Hailin Xu

**Affiliations:** 1 Orthopaedic and Traumatology, Peking University People’s Hospital, Beijing, 100044 China; 2 Orthopaedic, Qingdao Women and Children’s Hospital, Shandong, China; 3 Orthopaedic, Beijing Daxing District Hospital of Integrated Chinese and Western Medicine, Beijing, China; 4 Orthopaedic, Luanzhou People’s Hospital, Hebei, China

**Keywords:** Posterior malleolar fracture, Surgical fixation, Fracture morphology

## Abstract

**Background:**

The treatment of posterior malleolar fractures is changing rapidly, and the evidence base is still catching up. This study aimed to assess the mid-term prognosis of posterior malleolar fractures based on different morphological types and provides evidence for the treatment of posterior malleolar fractures.

**Methods:**

We retrospectively analyzed the data of inpatients with posterior malleolar fractures from 1 January 2012 to 31 December 2019 at one high-volume tertiary trauma center. Fracture morphology was classified into small-shell fragment, single-fragment (small-fragment and large-fragment) and multifragment (double-fragment and compressive-fragment) by computed tomography according to our previous study. All patients were followed up at an average of 5.06 (range, 2.21–8.70) years. The Olerud-Molander Ankle Score (OMAS), EuroQol-5 Dimensions (EQ-5D) and American Orthopedic Foot and Ankle Society (AOFAS) score were recorded.

**Results:**

Seventy-nine patients were included, and 7 patients were classified into the small-shell group, 52 patients into the single-fragment group and 20 patients into the multifragment group. Of all the patients, the average OMAS, EQ-5D and AOFAS scores were 85.9, 82.8 and 92.5, respectively. In the single-fragment group, patients who underwent surgical fixation in the posterior malleolus had significantly better scores (*P* = 0.037, 0.033 and 0.027). Among the patients with small fragments, the surgical fixation group also had higher OMAS (93.1 ± 7.5 vs. 83.5 ± 19.5, *P* = 0.042) and AOFAS scores (98.1 ± 3.1 vs. 91.0 ± 14.1, *P* = 0.028). The mean OMAS, EQ-5D and AOFAS scores were 85.5, 85.7 and 91.7, respectively, in patients with multiple fragments who underwent surgical fixation.

**Conclusion:**

This study shows that in fractures with a single fragment, surgical fixation of the posterior malleolar fragment led to a better prognosis in the midterm. All single fragments should be fixed regardless of size. Fixation of the posterior region in all single- and multi-fragments in posterior malleolar fractures led to satisfactory outcomes.

**Level of Evidence:**

Level III, follow-up study.

## Background

Ankle fracture is one of the most common bone and joint injuries and is associated with heavy economic burdens. Inappropriate treatment may cause severe complications such as checkrein deformities [1–4]. Posterior malleolar fractures are referred to the fractures involving the posterior rim of the distal end of the tibia and occur in up to 50% of all malleolus fractures [5].

The indication for internal fixation remains controversial in tri-malleolar fracture [6]. In the last century, the traditional view was that fragment areas involving more than one-third of the distal tibial articular surface should be fixed [7, 8]. This protocol has been challenged recently, as some researchers proposed that the treatment decision should be based on morphology rather than fragment area [9, 10]. However, further promotion of the clinical algorithm is limited due to the lack of evidence derived from long-term postoperative follow-up.

In our previous study, we applied the 3-dimensional computed tomography (CT) to describe the distribution of the posterior malleolar fracture lines and further related the anatomy of the posterior inferior tibiofibular ligament with the fragments [11]. The purpose of this study was to assess the functional outcomes of different morphology groups and provide mid-to-long-term evidence for posterior malleolar fracture treatment.

## Methods

### Subject

Patients with posterior malleolar fractures who received surgical interventions from 1 January 2012 to 31 December 2019 at one high-volume tertiary trauma center were enrolled. The exclusion criteria were listed as follows: 1. younger than 18 years old, 2. no CT scan, and 3. no surgical treatment. The clinical data of qualified respondents were retrospectively reviewed.

### Classification and surgical decision

**Fig. 1:**
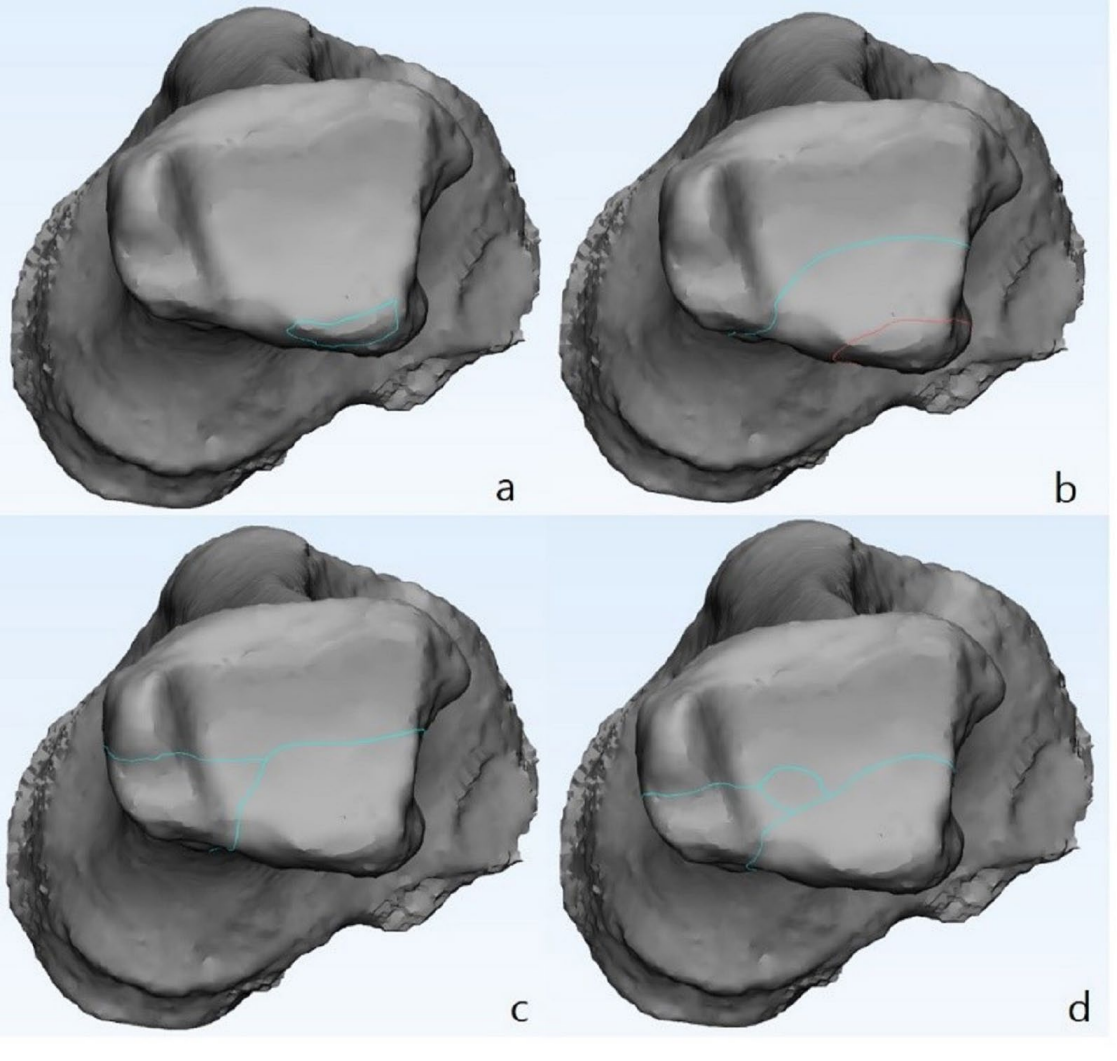
Classification of posterior malleolar fractures. **a** Small-shell fragment. **b** Single-fragment: The red line indicates the small-fragment, and the green line indicates the large-fragment. **c** Double-fragment. **d** Compressive fragment

The detailed new CT-based classification of posterior malleolar fractures proposed in our previous study is presented in Fig. 1 [11]. The CT scans were reviewed, and posterior malleolar fragments were classified into small-shell, single-fragment, and multifragment groups according to the mechanism of injury as proposed in our previous study. In this study, a small-shell fragment was distinguished from a single fragment by the fracture line involvement of only a few fibular notches and a small part of the posterior lip of the tibial plafond. The single fragment group included small-fragment and large-fragment, and in the transverse plane, the fracture line of the small-fragment originated from one-third of the entire tibialis posterior lip, whereas those in the large-fragment group originated from the groove of the tibialis posterior [11]. Multifragment referred to double-fragment and compressive-fragment. In the multifragment group, double-fragment included posterolateral and posteromedial parts and compressive-fragment included impacted fragments in the articular surface [11]. Surgical treatments of the medial malleolus and lateral malleolus were undertaken according to AO fixation principles, and the determination of whether fixation of the posterior malleolus was necessary was made after detailed preoperative discussion by experienced foot and ankle surgeons. Surgical treatments were performed when the soft tissue envelope allowed internal fixation. The intraoperative Cotton test and dorsiflexion external rotation stress test were performed to assess the syndesmosis stability. Further syndesmosis fixation was made with cortical syndesmotic screws or suture buttons when there was instability of syndesmosis [12]. In the case of intra-articular impacted fragments found on CT preoperatively, two surgeons decided to reduce or remove them based on the size of the fragment and experience.

### Surgical approaches

**Fig. 2:**
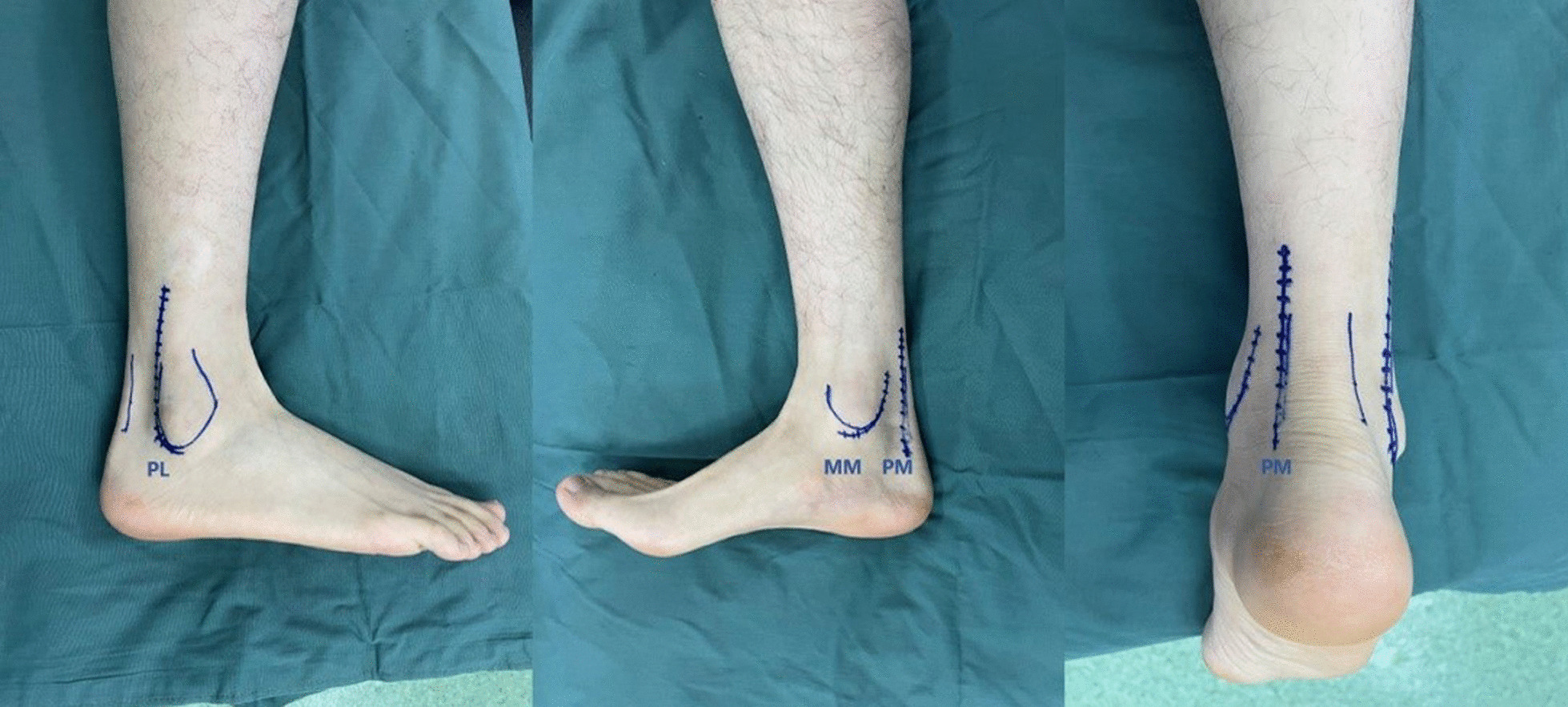
Lateral, medial and posterior views of the surgical approach. *PL* posterolateral, *PM* posteromedial, *MM* modified medial

According to our classification of posterior malleolar fractures, three different surgical approaches were adopted for optimal exposure and reduction in fractures under direct vision (Fig. 2).

For small-shell fragments, the posterior malleolus was not exposed. Lateral and medial malleolus fracture was fixed and inferior tibiofibular syndesmosis stability was restored.

For a single fragment, the posterolateral (PL) approach was recommended for small-fragment exposure. Accordingly, the modified medial (MM) approach was applied for large-fragment exposure. The incision of the PL approach was made near the posterior border of the fibula as previously reported [13, 14]. The incision of the MM approach was made along the posterior medial border of the distal tibia and later curved forward. After the tibialis posterior tendon sheath was opened, the tibialis posterior was retracted anteriorly and medially, and the posteromedial tibia was exposed. The fragment in the anterior colliculus of the medial malleolus can also be fixed anterior to the tibialis posterior through this approach.

For multiple fragments, we chose the posteromedial (PM) approach. An incision was made along the medial border of the Achilles tendon. After the investing fascia was opened and flexor hallucis longus was exposed, blunt dissection was performed, with fingers or curved forceps, and the posterior border of the distal tibia was fully exposed between the neurovascular bundle and flexor hallucis longus. The other method was to use the PL and MM approach. The PL approach was reasonable for the posterolateral fragments and the MM approach made it possible to fix the posteromedial fragments involved in the medial malleolus.

### Postoperative management and statistical analysis

Active flexion and extension exercises of knees and toes were started the first day after surgery and ankles were fixed with braces and were immobilized for 2 weeks. Then passive flexion and extension training of the ankles was started under the guidance of a rehabilitation physician. Active flexion and extension exercises of the ankles were started 4 weeks post-surgery. Patients without tibiofibular fixation began partial weight-bearing at 6 weeks and full weight-bearing at 8 weeks post-operatively. Patients who underwent tibiofibular fixation with syndesmosis screws started weight-bearing at 12 weeks after the syndesmosis screws were removed. Patients who underwent tibiofibular fixation with suture buttons started weight bearing 8 weeks after surgery.

Radiographic imaging and functional examinations of the ankle were performed at 4, 8, and 12 weeks, 6 months, and 1 year post-operatively. All patients were followed up for an average of 5.06 (2.21–8.70) years. The Olerud-Molander Ankle Score (OMAS), EuroQol-5 Dimensions (EQ-5D) and American Orthopedic Foot and Ankle Society (AOFAS) score were recorded.

Continuous variables are described as the mean ± s.d. (standard deviation). Categorical variables are presented as frequencies (percentages). Student’s t test was chosen as the hypothesis test of differences in scale scores between subgroups of patients. Categorical variables were analyzed with the chi-square test or Fisher’s test. A value of *P* < 0.05 was considered statistically significant. All statistics were conducted using SPSS Statistics 20 (IBM, Armonk, NY).

## Results

A total of 102 patients met the inclusion criteria and 23 patients were excluded due to loss to follow-up. Finally, seventy-nine patients completed the questionnaires and were successfully enrolled for further analysis. Baseline information is shown in Table 1.

**Table 1:** Characteristics of patients

	Value
Sex (%)	
Male	25 (31.6)
Female	54 (68.4)
Age, year (%)	
≥60	25 (31.6)
<60	54 (68.4)
Lauge-Hansen classification (%)	
SER	63 (79.7)
PER	13 (16.5)
PA	3 (3.8)
SA	0
Fragment classification (%)	
Small-shell fragment	7 (8.9)
Small fragment	37 (46.8)
Large fragment	15 (19.0)
Double fragment	8 (10.1)
Compressive fragment	12 (15.2)
Average follow-up time, y (range)	5.06 (2.21–8.70)

*PA* pronation-abduction, *PER* pronation-external rotation, *SER* supination-external rotation, *SA* supination-adduction, *s.d.* standard deviation

There were 7 patients in the small-shell group, 52 patients in the single-fragment group and 20 patients in the multifragment group. In the small-fragment group, there were 24 (64.9%) patients who did not undergo fixation of the posterior malleolus. Ten patients underwent fixation of syndesmosis using suture buttons (5 patients) and cortical screws (5 patients). In the large-fragment, double-fragment and compressive-fragment groups, only 1 patient underwent conservative treatment of the posterior malleolar fragment. This patient had poor OMASs, EQ-5Ds, and AOFAS scores (55.0, 70.0, 58.0, respectively) compared with the average level.

**Fig. 3:**
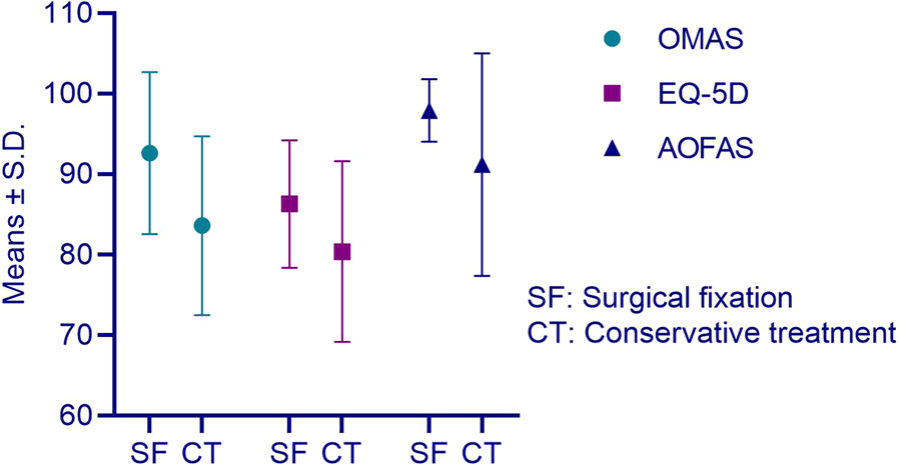
OMASs, EQ-5Ds and AOFAS scores in patients with single fragments

The OMASs, EQ-5Ds, and AOFAS scores of the different groups are presented in Table 2. The patients with compressive fragments had the lowest OMAS and AOFAS scores. The patients with a single fragment in the posterior malleolus were further divided into conservative and surgical treatment subgroups. Patients who underwent surgical fixation had a significantly better prognosis than those who underwent conservative treatment significantly (Fig. 3). Furthermore, among the patients with small fragments, the surgical fixation group also had higher OMASs (93.1 ± 7.5 vs. 83.5 ± 19.5, *P* = 0.042) and AOFAS scores (98.1 ± 3.1 vs. 91.0 ± 14.1, *P* = 0.028). If only the size of fragments was compared, between the large- and small-fragment groups, there was no significant difference in the OMASs (91.8 ± 6.8 vs. 86.6 ± 5.6, *P* = 0.300), EQ-5Ds (86.9 ± 5.2 vs. 82.2 ± 3.4, *P* = 0.146) and AOFAS scores (97.5 ± 3.6 vs 93.5 ± 3.9, *P* = 0.099). In the case of multiple fragments, a significant difference was found in the OMASs between the double-fragment and compressive fragment groups (92.5 ± 6.3 vs. 80.8 ± 8.3, *P* = 0.036).

**Table 2:** OMAS, EQ-5D and AOFAS scores of different groups

	OMAS (s.d.)	EQ-5D (s.d.)	AOFAS (s.d.)
Small shell	84.3 (14.7)	81.4 (11.2)	92.1 (9.3)
Small fragment	86.6 (16.6)	82.2 (10.1)	93.5 (11.7)
Large fragment	91.8 (11.4)	86.9 (8.8)	97.4 (4.5)
Double fragment	92.5 (7.1)	87.5 (6.1)	95.3 (4.4)
Compressive fragment	80.8 (12.6)	84.5 (9.9)	84.5 (13)
Total	85.9 (14.9)	82.8 (9.9)	92.5 (10.6)

*s.d.* standard deviation

## Discussion

This research provided mid-term clinical outcomes of posterior malleolar fracture treatment under morphology guidance and revealed some key points in the treatment of posterior malleolar fracture.

For a long time, orthopedists were accustomed to making decisions based on fragment size. However, paradoxical results have often been reported. Previous literature pointed out that the estimated ratio of the fragment area to the articular surface is 14.96%, which means that most of the single fragments are less than 25% or one-third of the articular surfaces [15]. This indicates that surgical fixation was not necessary in most single-fragment cases. Some researchers have described that conservative treatment of posterior malleolar fractures results in good clinical and radiological outcomes [16, 17]. In contrast, others found that surgical fixation of fragments < 25% of the articular surface had great outcomes in the short term [18]. Neglecting the morphology and ligament attachment, merely analyzing prognosis according to size may be the main reason for these conflicting conclusions.

Our study demonstrated that, to achieve a better prognosis, surgical fixation of a single-fragment in the posterior malleolus was significant superior to conservative treatment. In the study of McHale, when the fragment size was 10–20%, the clinical outcome was worst because this group was mostly treated without direct fixation [19]. In our study, 64.9% of the patients with small-fragment fractures did not undergo fixation of the posterior malleolus. This demonstrated that the small-fragment in the posterior malleolus did not receive sufficient attention. Fixation of the posterior malleolar fragment was able to restore syndesmosis stability and led to improved clinical outcomes [20, 21]. Recent studies have revealed that morphology rather than size should be considered when determining the treatment of posterior malleolar fractures [9, 22–24]. Our previous study on the distribution of posterior malleolar fracture lines also classified patients according to the fracture line distribution rather than fracture area [11]. In this study, by comparing prognosis between fixation and nonfixation in patients with small fragments in the posterior malleolus, we also provided evidence that surgical fixation of posterior malleolar fragments was more advantageous. As shown in Fig. 3, surgical fixation of a single fragment had an advantage over conservative treatment with regard to quality of life preservation and improved prognosis. This is reasonable considering the posterior inferior tibiofibular ligament avulsion mechanism we proposed in our previous study [11]. Therefore, we supposed that in posterior malleolar fracture, the morphology of the fragment was demonstrated to be more significant than fragment size.

According to our study, the compressive-fragment is a predicting factor for low OMAS scores. Although surgeons have attached great importance to the reduction in intra-articular fragments, some fragments still have to be removed. This may lead to an uneven articular surface which is associated with a poor prognosis [25]. In addition, although there was no significant difference in the OMASs between the small-, large- and double-fragment groups, it is interesting that the average score in the small-fragment group was lower than that in the other two groups. Perhaps the high proportion of patients who received conservative treatment in the small-fragment group was one of the factors. However, this result also indicated that all fractures with multiple fragments should undergo surgical fixation, which corresponds to a more favorable prognosis.

**Fig. 4:**
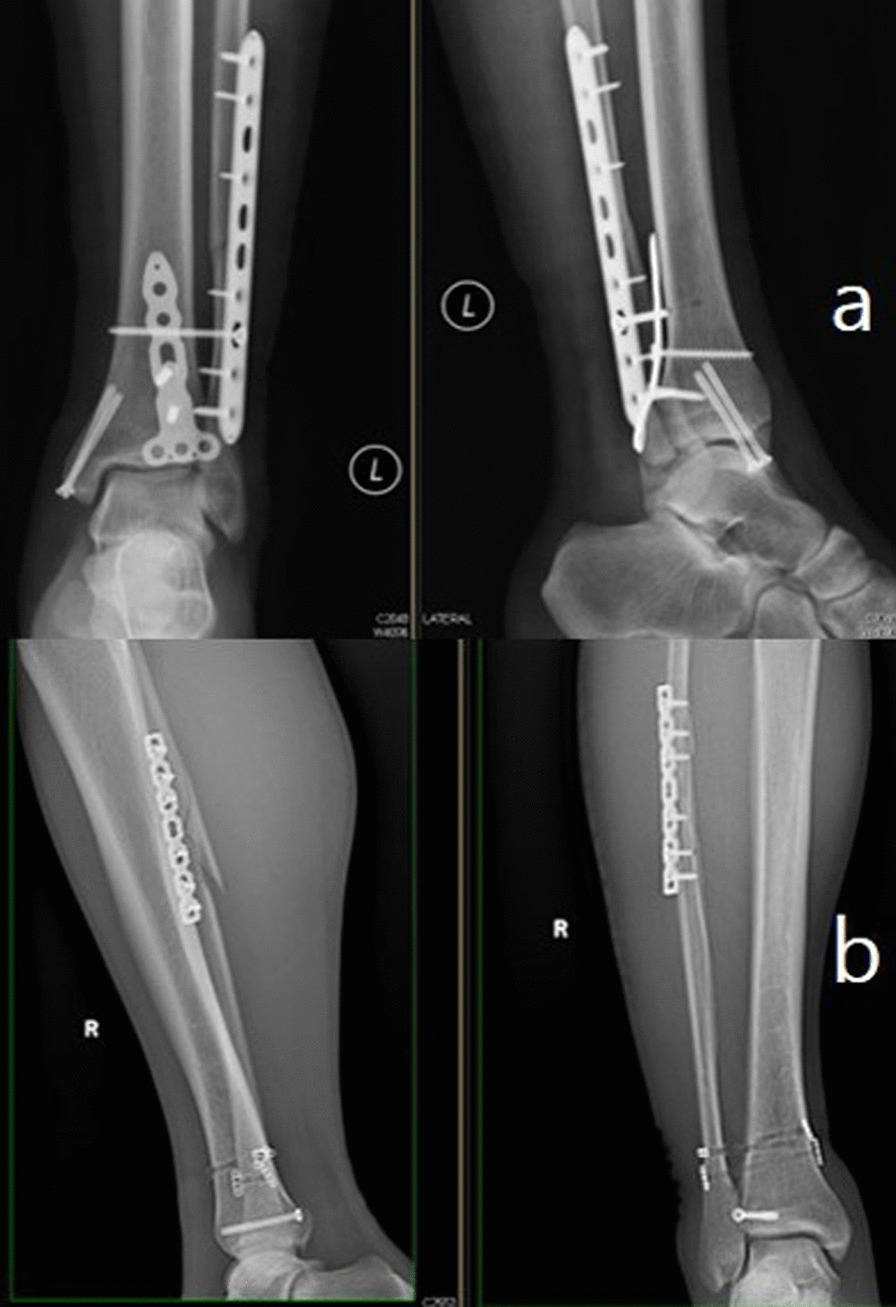
Two patients underwent both posterior malleolar fragment and syndesmosis fixation. The first patient underwent plate fixation of the posterior malleolus fragment and screw fixation of the syndesmosis (**a**). The other patients underwent screw fixation of the posterior malleolar fragment and suture button fixation of the syndesmosis (**b**)

Mason also performed a 2-year follow-up according to the Mason classification which depends on the mechanism of ankle fracture [26]. All the patients with Mason type 1 fracture underwent syndesmotic reduction and fixation, compared with only 2 (33%) patients in our study. As we conducted a dorsiflexion external rotation stress test and lateral distraction of the fibula intraoperatively, not every patient with a small-shell fragment had syndesmosis instability. In addition, after fixation of the posterior malleolar fragment, the syndesmosis still needed to be fixed in 7 patients in our study, 3 and 4 of whom were classified as having pronated external rotation and supinated external rotation, respectively. The X-rays of the 2 patients are shown in Fig. 4. This finding indicated that interosseous ligament injury is a significant risk factor in syndesmosis fixation. There were also a few differences found when comparing the surgical approaches [27, 28]. In the PM approach, we exposed the posterior malleolus between the neurovascular bundle and flexor hallucis longus. We believe that the posteromedial fragment can be observed more clearly in this manner. In addition, in a Mason type 2A fracture, when the Volkmann fragment is large, the PL approach may not be able to expose the entire fragment. The PM approach was better in this situation.

We acknowledge that our study has several limitations. First, this study is a retrospective study, and all treatment decisions were made by surgeons with no consensus. The criteria for choosing conservative or surgical treatment may vary, and the follow-up period may have an impact on the study accuracy. Second, regarding patient characteristics, most of our patients were female, with a lower participation in intensive sports, which may have led to the relatively higher subjective scores related to prognosis. Third, fixation of the posterior malleolar fragment may be performed by screws or plates according to the preference of the surgeons. This may also lead to different results in mid-term prognosis. In future, a continuous questionnaire should be administered to patients with mid-to-long-term follow-up, and a protocol for postoperative rehabilitation should also be developed.

## Conclusion

This study shows that in fractures consisting of a single fragment, surgical fixation of the posterior malleolar fragment led to a better prognosis in the mid-term. All single fragments should be fixed regardless of size. Fixation of the posterior region in all single- and multi-fragments in posterior malleolar fractures led to satisfactory outcomes.

## Data Availability

The datasets used and analyzed during the current study are available from the corresponding author on reasonable request.

## Abbreviations

OMAS: The Olerud-Molander Ankle Score
EQ-5D: EuroQol-5 dimensions
AOFAS: American Orthopedic Foot and Ankle Society
PL: Posterolateral
PM: Posteromedial
MM: Modified medial
s.d.: Standard deviation
